# Fast response paper based visual color change gas sensor for efficient ammonia detection at room temperature

**DOI:** 10.1038/s41598-018-33365-3

**Published:** 2018-11-15

**Authors:** Avisek Maity, Barnali Ghosh

**Affiliations:** 1 Department of Condensed Matter Physics and Materials Sciences, S.N. Bose National Centre for Basic Sciences, JD Block, Sec-III, Salt Lake, Kolkata, 700106 India; 2 Technical Research Centre (TRC), S.N. Bose National Centre for Basic Sciences, JD Block, Sec-III, Salt Lake, Kolkata, 700106 India

## Abstract

We show that a cheap, disposable type rapid paper sensor (working at room temperature) can be made using perovskite halide CH_3_NH_3_PbI_3_ (MAPI) to detect presence of the toxic ammonia gas (NH_3_)by just color change, where the black colored MAPI film (on the paper) changes to yellow color in presence of a very low concentration of NH_3_ gas. The sensor can detect presence of NH_3_ gas in open or closed atmosphere down to around 10 ppm with a response time of nearly 10 sec which decreases to few seconds when the concentration exceeds 20 ppm. The easy to fabricate sensor paper being a visual sensor does not need any other extra equipment for its operation. The sensor is not sensitive to moisture with RH upto 90% and does not also respond to gases like Methane (CH_4_), Nitrous Oxide (N_2_O), Carbon dioxide (CO_2_) etc in the test chamber each up to a concentration of 500 ppm. Conversion/decomposition of MAPI to PbI_2_ on exposure to NH_3_ has been proposed as the mechanism of color change and the mechanism has been established using a collection of techniques like XRD, EDX, UV-Visible absorption and Photo Luminescence.

## Introduction

Realization of thin film gas sensors for efficient and cost-effective detection of toxic gases is a topic of considerable current interest^1–3^. The reliability of these types of sensors has great impact and relevance in issues where hazardous gases can be detected efficiently in such environments that involve deployment of human resources. Most of the thin film based gas sensors reported earlier are electrical signal based and would need a peripheral arrangement for detection of the gas^4–6^. It is envisaged that if a color change sensor can be made where a visual detection can detect the hazardous gas in ppm level that would make it extremely easy to use as well as cost effective as it would need no electronics peripheral as well as need of trained operator can be obliterated and it will also be maintenance free. As examples, color change sensors based on papers are widely used for pH measurements as well as for measurements of glucose level in urine^7^. However, such easy to use sensors are not available for hazardous gases till date. In this paper, we report such a color change visual sensor based on simple paper, where exposure to ammonia can be detected quickly at room temperature with high selectivity as well as sensitivity (e.g. down to 10 ppm). The sensor being cheap is disposable and the ready to use sensor can be suitably preserved for long term shelf-life (at least 6 months). Since the sensor needs no electrical read–out and no external peripherals, it is field useable without need for trained operators. The innovation involves effective utilization of new material like perovskite halide which has not been utilized before for effective gas sensing.

One of the most hazardous environmental pollutants in the atmosphere is ammonia (NH_3_). Detection of the presence of NH_3_ at a low level is most desirable as the presence of the gas can occur in several areas like refrigeration, food processing and storage, fertilizers, environmental protection, chemical technology, ammonification by nitrogen cycle etc. Generally, an acceptable level of NH_3_ gas is 8-h exposure limit at 25 ppm and a short-term (15 min) exposure level at 35 ppm^4–6,8^. Sustained exposure may cause severe problem on human health. It can even be fatal if the inhaled gas has NH_3_ above an acceptable limit of 500 ppm for 30 minutes^8^. It is thus envisaged that development of a practical low cost, visual gas sensor (like a paper) for rapid and selective detection of atmospheric NH_3_ gas in open as well as closed environment will be of great importance as personnel working in these areas can quickly detect even low level ammonia.

Up to now most NH_3_ sensing materials are metal-oxide semiconductor systems that are based on electrical sensing or optical detection^9–12^. However, the high working temperature (200–500 °C), low selectivity, and slow response/recovery rate and poor detection limit of metal-oxide-based sensors stand as a road-block to most practical applications. In that context investigation of new materials for NH_3_ detection at room temperature with high sensitivity and selectivity will be an important innovative step forward.

In this paper we have taken the approach to deviate from standard oxide materials used for NH_3_ gas detection. We show that perovskite halide (CH_3_NH_3_PbI_3_) based thin film sensor fabricated on a paper via simple and cost effective solution growth process can detect NH_3_ gas by simple color change effect without any external detection instrument. The visual gas sensor is highly selective to NH_3_ gas only and excellent response and recovery time and has a very low detection limit. Moreover, it needs no heating for its operation and thus needs no external power supply.

Hybride halide perovskite especially Methyl Ammonium Lead Iodide (CH_3_NH_3_PbI_3_) or MAPI is widely used material for photovoltaics^13^. But as an active material in gas sensors this material has not been investigated. Some previous works have been reported about the color change of the perovskite halide (MAPI) in presence of NH_3_^14,15^. However the papers did not mention the concentration of NH_3_ used and no definitive explanation was given about proper mechanism of the color change. Novelty of the paper is that it uses certain properties of MAPI related to its lack of stability to NH_3_ to make it work as a visual sensor. It also innovates a way to grow this material on a porous material like a paper so that a cheap and disposable sensor can be made. The present report through a series of tests establishes the mechanism of the color change sensor operation.

## Results

### Ammonia Gas Sensing Property of MAPI Coated Paper

**Figure 1:**
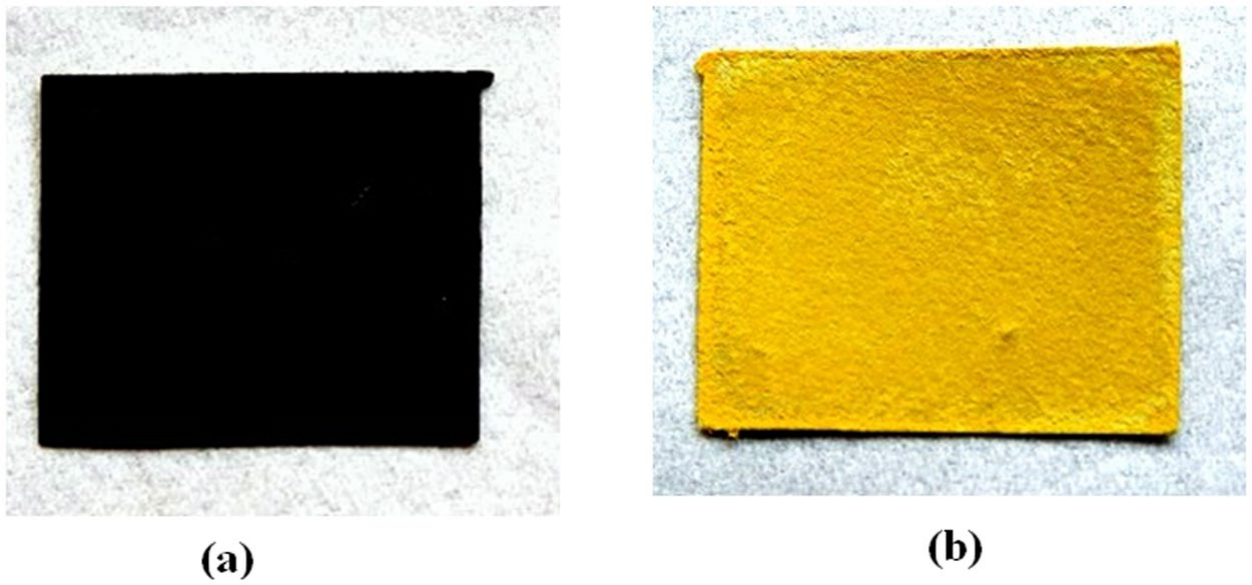
Color change response of the MAPI Sensor: (**a**) Represents the original black color of the unexposed film. (**b**) Represents the yellow color of the film in presence of NH_3_ gas.

The gas sensing property of the NH_3_ sensor paper was investigated by simple visual color change method in a test chamber that allows controlled gas environment. The initial test condition was set up by purging the test chamber by flowing ambient Nitrogen for 15 minutes. On exposure to NH_3_ gas the sensor paper changes color from black to yellow as shown in Fig. 1. All sensing measurements were carried out at room temperature.

It is also noteworthy that the color of the MAPI film changes from black to yellow in presence of NH_3_ gas both in open atmosphere and as well as closed test chamber with suitable injection of NH_3_.

**Figure 2:**
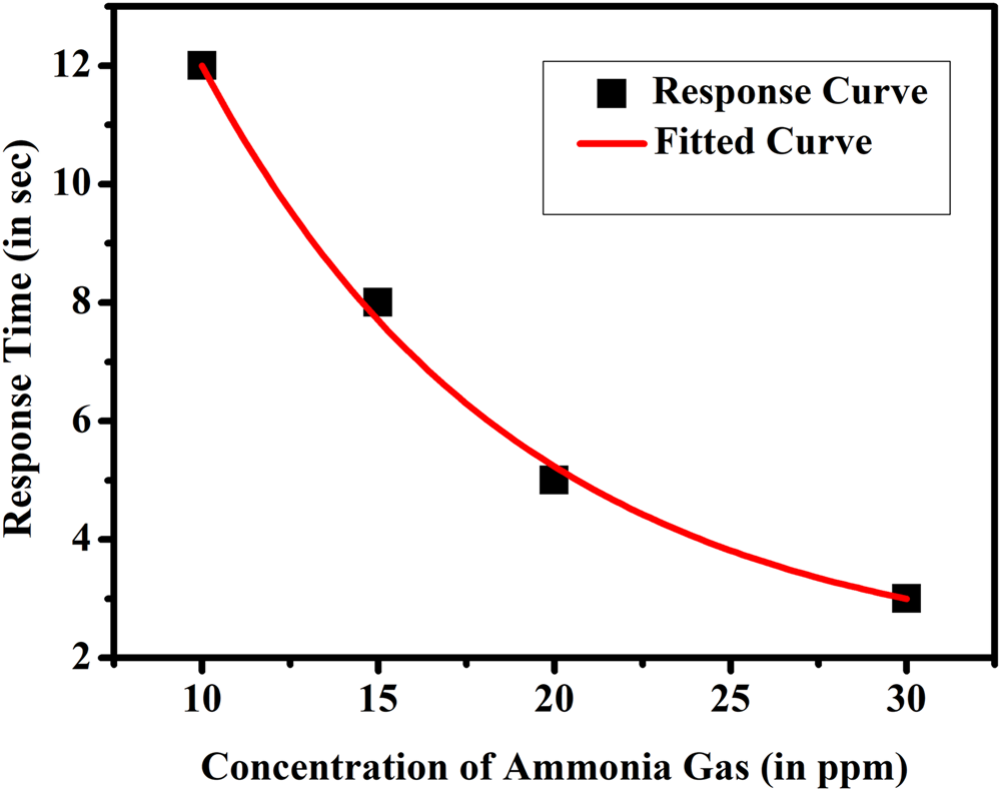
Dependence of the time response of the paper sensor to different concentrations of NH_3_ gas at room temperature.

We investigated the time of response for color change at different NH_3_ gas concentration. This was done by injecting NH_3_ gas in the test chamber and recording the experiment in a video camera. From video we got the necessary response time to change the color of the film from black to yellow. Figure 2 shows the dependence of color change response time τ of the MAPI paper sensor for different gas concentrations. It can be seen that for gas concentration of only 10-ppm the visual sensor can change color within a response time of around 12 sec. Bare paper did not show any response of color change when exposed to NH_3_ gas. Also below 10 ppm concentration of NH_3_ gas, the color change effect did not occur. It is clearly observed as shown in Fig. 2 that the MAPI sensor exhibits a faster response when exposed to higher concentrations of NH_3_ gas.

**Figure 3:**
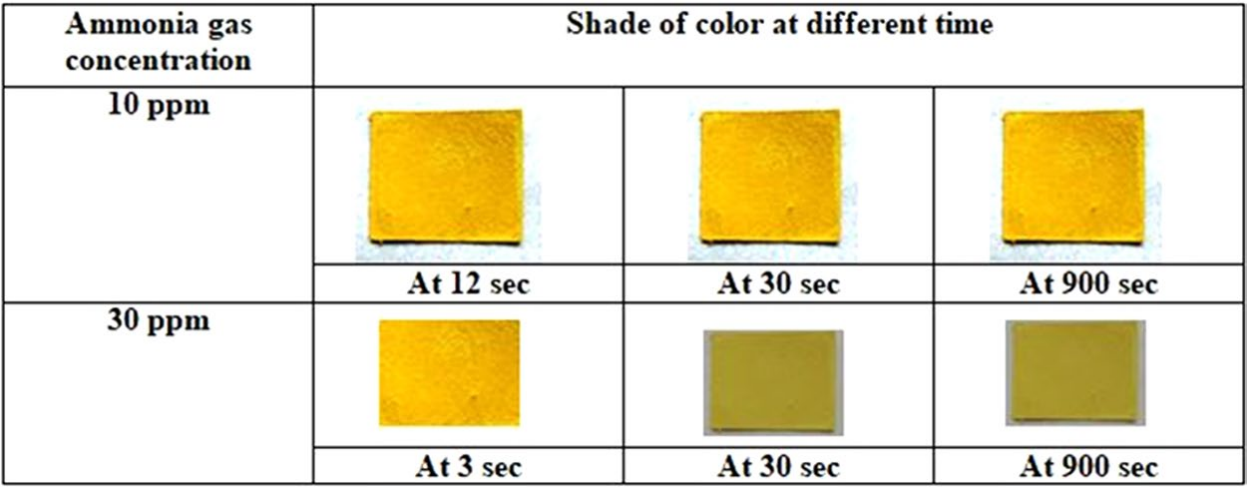
Color of the paper sensor at different NH_3_ concentrations for long time exposure.

The response time τ shows an exponential dependence on concentration c such that, ~exp(−*c*/*c*_0_) (*c*_0_ being a constant) so that with increase of the NH_3_ concentration the response time quickly decreases. We have also recorded the different color map of the sensor exposed at different concentrations of NH_3_ gas. The color of the sensor changes subsequently with respect to different ppm level. The shade of the yellow color changes with higher concentration. It has been observed that for higher concentration (30 ppm) the yellow color has a shade and dark yellow changes to pale yellow for a long exposure as shown in Fig. 3. However the structure in both the yellow color is same (PbI_2_).Between 10 to 30 ppm the difference of shade of yellow color is not distinguishable visually.

As a visual sensor to assess the immediate extent of danger of presence of the NH_3_ in an ambience, this is a desirable feature. If the concentration is low the sensor takes nearly 10 secs to respond and this will not lead to detrimental exposure. On the other hand when the concentration is relatively high (~20–25 ppm) and it approaches a danger level, the senor quickly turns color within 5 sec and gives a visual warning. For any operator in a hazardous environment this will give an immediate danger signal.

The response time is very fast compared to reported NH_3_ sensor using other materials. A table is given in Supplementary data (Table S1) where it can be seen that the response time for these materials lie above 100 sec and may even be more than 1000 sec in some cases. The visual sensor reported here thus can be considered to have a much faster response. The commercially available NH_3_ sensors based on electrical read outs has typically response time of ~2–3 min (for 25–30 ppm NH_3_ concentration). Also it needs electrical power to heat up the sensing element (SnO_2_) with proper electronic circuits.

### Selectivity of The Paper Sensor to Ammonia Gas

Selectivity is one of the important properties of a gas sensor. Selectivity of the visual sensor was tested by adding other hazards gases like Methane (CH_4_), Nitrous Oxide (N_2_O), Carbon dioxide (CO_2_) etc in the test chamber each up to a concentration of 500 ppm for sufficiently long time (~15 minutes or more). No significant visual color change had been observed in all these cases. This strongly suggests the visual gas sensor is highly selective to the NH_3_ gas only. The specific mechanism of color change is suggested below. NH_3_ decomposes MAPI and this does not occur with other gases. This ensures that the paper sensor has high level of selectivity towards NH_3_.

### Effect of Humidity

**Figure 4:**
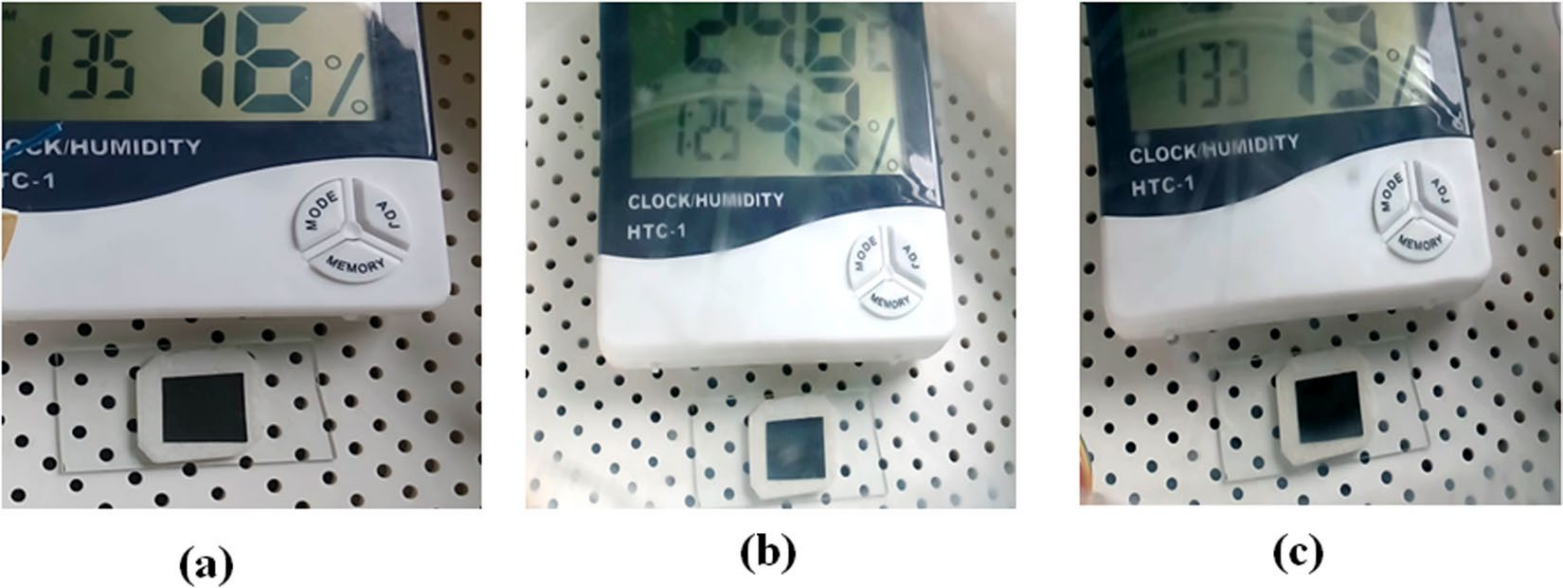
Photograph of paper sensor in atmosphere of varying humidity as shown by the RH meter reading: (**a**) At relative humidity 76% (**b**) At relative humidity 43% (**c**) At relative humidity 13%.

Stability of MAPI coated paper towards exposure to moisture is an important parameter for its usability in an open atmospheric condition where there may be a likelihood of the moisture affecting the sensor. The effect of humidity on MAPI sensor was investigated in a controlled way by subjecting the sensor paper to atmosphere in the test chamber with different percentage of relative humidity (% RH). It was observed that no color change occurred that results from exposure to humidity for RH range from 10% to 90%. In Fig. 4 we have shown photographs of the sensor paper when exposed to three relative humidity (13%, 43% and 76%) as displayed by the RH meter. The pristine black color is maintained irrespective of the relative humidity (exposure time ~180 sec). In humid condition the sensor can response to visual color change when the sensor paper is exposed to NH_3_ gas only or aqueous NH_3._ However humidity alone cannot change the color.

### Stability Towards Storage and Shelf-life

**Figure 5:**
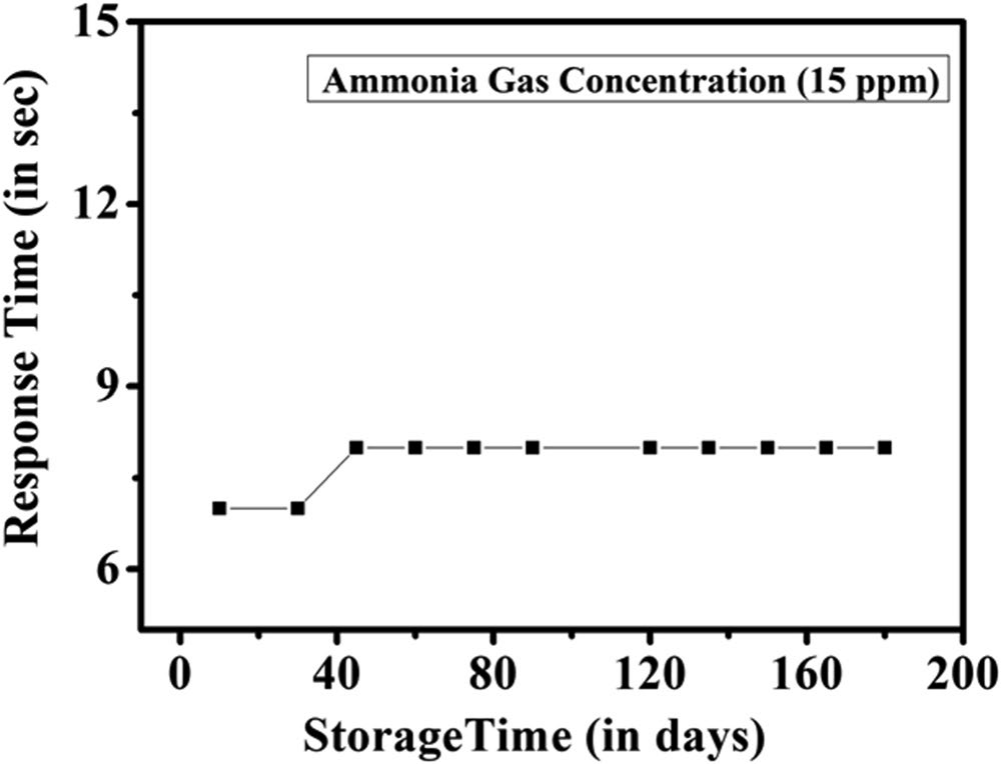
Stability of the sensor against long term storage. Sensing study was performed at room temperature upon exposure to NH_3_ gas of fixed concentration 15 ppm.

A collection of sensor papers were stored in a desiccator. Temperature range tested between 20 °c to 35 °c for storage as well as for experiment. At interval of 15 days one strip was taken out and then exposed to only 15 ppm of NH_3_ gas in the test chamber and response time was measured. This was continued for 180 days. The data are shown in Fig. 5. It can be seen that the response time; i.e the respective time needed to change the color of the sensor (from black to yellow) of the paper sensor leading to visual color change is more or less constant after an initial small change (that occurs within about first 40 days). This small change in the response time however, does not affect its utility. The relatively high shelf -life and almost constant sensing performance of the sensor can be concluded that MAPI can stand as stable sensor for NH_3_ gas at room temperature.

To summarize the main results we find that MAPI coated sensor paper is a sensitive and it is an easy to use sensor that can detect very low concentration of NH_3_ gas (~10 ppm) visually just by its color change from black to yellow. We find the sensor is highly selective towards NH_3_ gas (as well as aqueous NH_3_) and is stable towards moisture. The ability of the cheap easy to make paper based MAPI sensor to trace low level of NH_3_ at room temperature with high selectivity and high sensitivity without any added electronics or need of any extra gadget, will have big impact in application and is of great practical relevance in areas of application where quick sensing is needed that no undue exposure will occur to NH_3_.

## Discussion

We propose that the mechanism of color change in the MAPI coated paper is due to degradation of MAPI to yellow colored PbI_2_. Below we establish this hypothesis through a set of experiments involving structural as well as spectroscopy tools that the end product after the color change is indeed PbI_2_.

**Figure 6:**
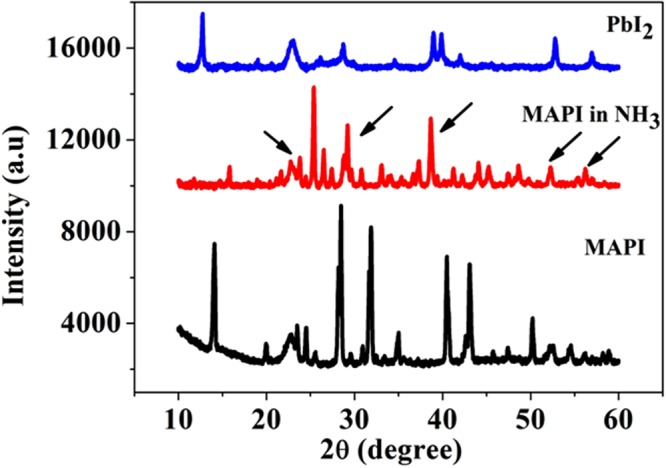
Comparison among XRD of unexposed pristine MAPI film on paper, exposed MAPI (to NH_3_) and PbI_2_ film on paper.

In order to detect any structural change of the MAPI film in presence of NH_3_, we also performed the XRD of the NH_3_ exposed MAPI film on the paper. Figure 6 shows the comparison between XRD patterns of the unexposed MAPI film and NH_3_ exposed MAPI film and also the XRD pattern of a PbI_2_ film grown on paper. The XRD pattern of the MAPI film changes substantially after the film is exposed to the NH_3_ gas. It can be seen from Fig. 6 that the XRD pattern of the gas exposed MAPI becomes comparable to that seen in the PbI_2_ film and most of the peaks of the two XRD pattern (marked by arrow) match. This establishes the identity of the new phase obtained from MAPI after NH_3_ exposure.

**Figure 7:**
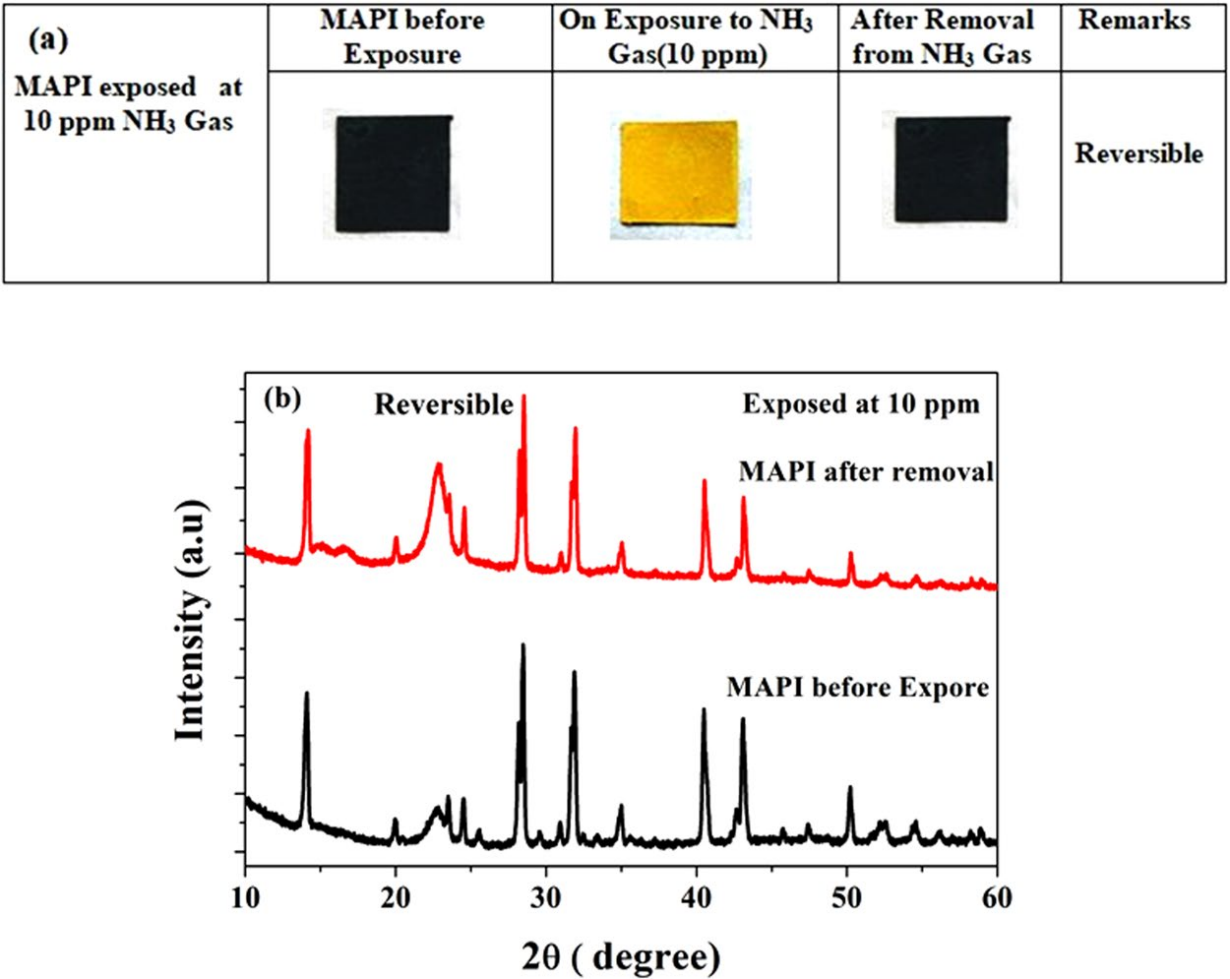
Exposure of MAPI to 10 ppm concentration of NH_3_ gas: (**a**) Photograph of MAPI before and after removal and (**b**) corresponding XRD pattern shows reversible nature.

It is noteworthy here that at low concentration (~10 ppm), the effect of color change is reversible and spontaneously returns back to MAPI (black colored) as shown in Fig. 7(a). Also from XRD pattern as shown in Fig. 7(b) indicates that after removal from NH_3_ gas, it returns back to the same crystallographic structure of MAPI.

**Figure 8:**
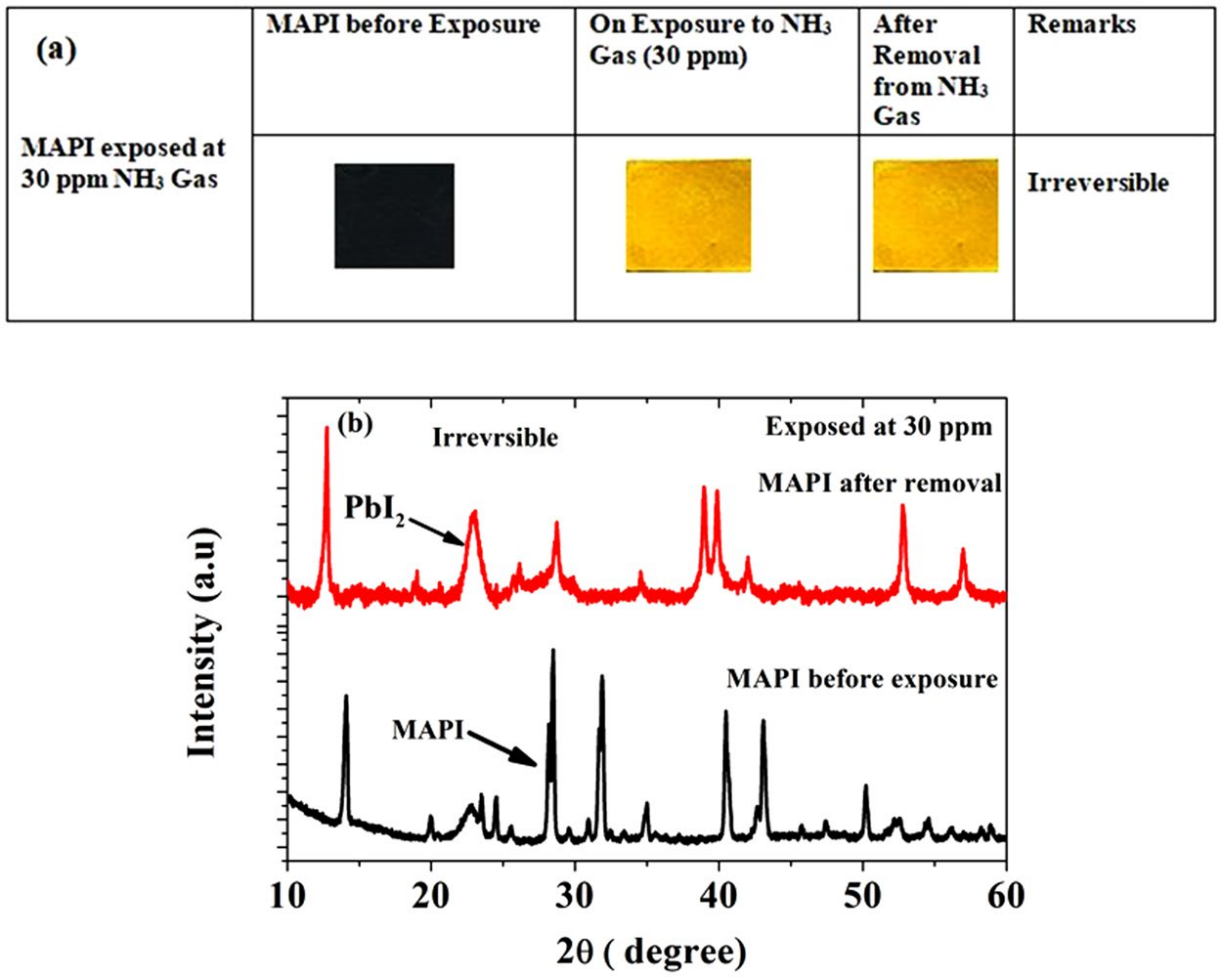
Exposure of MAPI to 30 ppm concentration NH_3_ gas. (**a**) Photo graph of MAPI before and after removal and (**b**) corresponding XRD pattern shows irreversible nature.

Whereas, for higher concentration (~30 ppm) the color changes on NH_3_ exposure to yellow colored film and it decomposes completely to PbI_2_ and unable to recover to MAPI spontaneously, hence it is irreversible, the photo graph is shown in Fig. 8(a). XRD pattern shows that MAPI has been fully decomposed to PbI_2_, plotted in Fig. 8(b).

As the sensor is grown on a paper; low cost synthesis and also the irreversibility issue observed from experimental observation makes the sensor disposable. Hence, the sensor can be used as ‘use and throw’ basis in cost effective manner like a pH paper.

We found that at higher concentration the time taken to complete the reaction from MAPI to PbI_2_ (yellow) is 2–4 secs, and makes the sensor irreversible.

In following sections we will discuss further about decomposition of MAPI to PbI_2_ as a proof of color change via different evidences.

**Figure 9:**
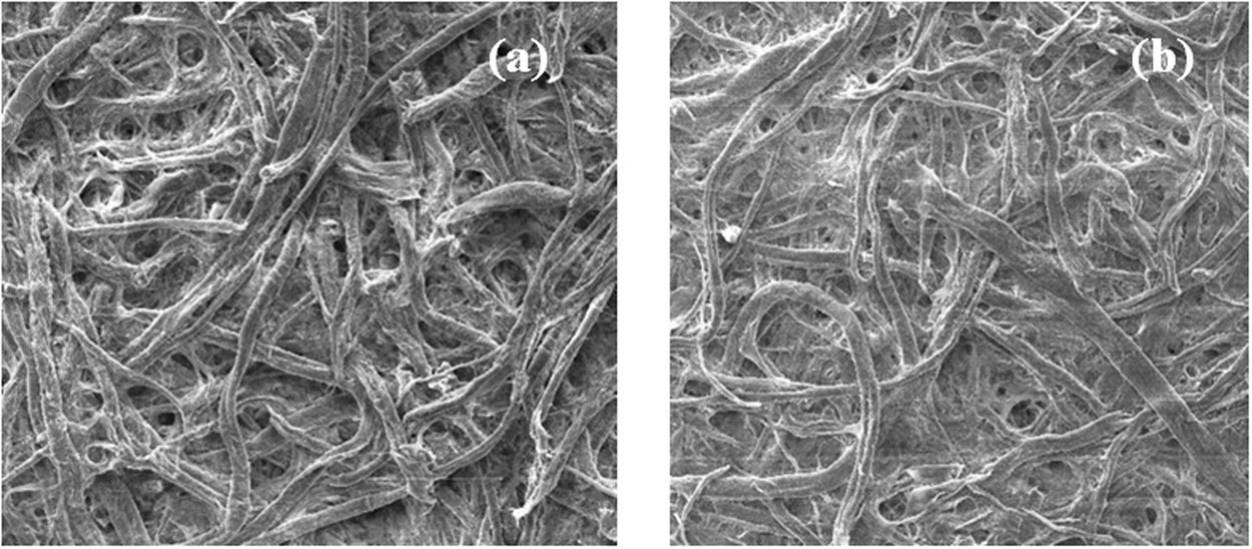
Representative FESEM images of (**a**) PbI_2_ coated and (**b**) MAPI in NH_3_ exposed condition.

Representative FESEM micrographs of lead iodide (PbI_2_) coated paper and that of the exposed MAPI film in NH_3_ environment are shown in Fig. 9(a) and 9(b) respectively. The SEM micrograph of MAPI coated paper has been shown in the experimental section. While the original MAPI film showed clear nanorod like structure grown on the fibers of the paper, on exposure to gas the morphology of the MAPI coated paper becomes fibrous similar to that of the bare paper as shown in experimental section. This also similar to the morphology of PbI_2_ coated paper as shown Fig. 9(a). The conversion of morphology of the MAPI coated paper on exposure to NH_3_ gas constitutes another proof that the formation of PbI_2_ on exposure to NH_3_ lead to color change.

**Figure 10:**
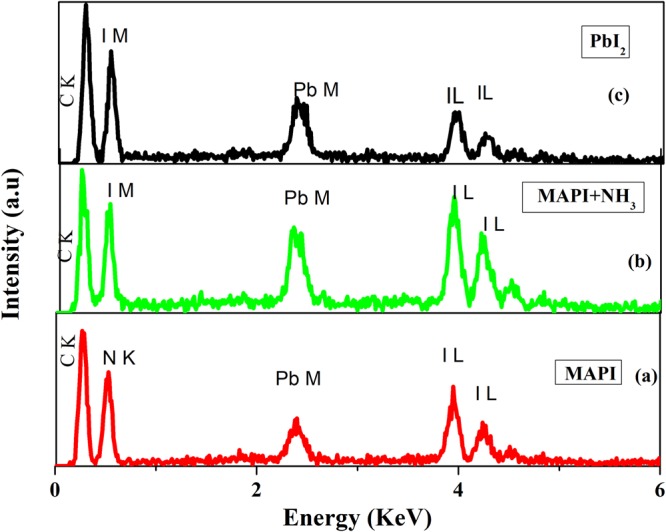
Comparison among EDS data of (**a**) Unexposed pristine MAPI on paper, (**b**) Exposed MAPI and (**c**) PbI_2_ film on paper.

In order to determine the changes in the chemical compositions of /constitute elements of the bare MAPI film on exposure to NH_3_ we performed energy dispersive X-ray analysis (EDX) of the as grown MAPI film and the gas exposed MAPI film in an FESEM. We also did the same measurement on the PbI_2_ film. We have used the atomic ratio $(\frac{Pb}{I})$ (=ratio of intensity of Pb and intensity of I (L line) as obtained from the EDX as a semi-quantitative parameter for comparison of the three films. (We note that the EDX measurements may not give a good quantitative estimate of the atomic fractions but is a semi-quantitative guide). The EDX data for the three films are shown in Fig. 10. The numbers obtained from the main lines are collected in Table 1. It was observed the $(\frac{Pb}{I})$ ratio increases in case of NH_3_ exposed film in comparison to the pristine MAPI film and it is close to the value of the $(\frac{Pb}{I})$ ratio in the PbI_2_ film. In the NH_3_ exposed MAPI film as well as the PbI_2_ film there is no presence of Nitrogen line, which is present in the pristine unexposed film. The large Carbon content arises from the cellulose in the paper. The EDX data provides additional confirmation that the MAPI film on gas exposure becomes PbI_2_.

**Table 1:** Comparison of $(\frac{Pb}{I})$ ratio among MAPI, NH_3_ exposed MAPI and bare PbI_2_ film.

Material	C^*^	N	I	Pb	Pb/I
MAPI	86.76	10.73	2.49	1.03	0.413
MAPI in NH_3_ gas	94.12	0	4.06	1.82	0.448
PbI_2_	97.34	0	1.84	0.80	0.434

^*^C content arises from cellulose of the paper.

**Figure 11:**
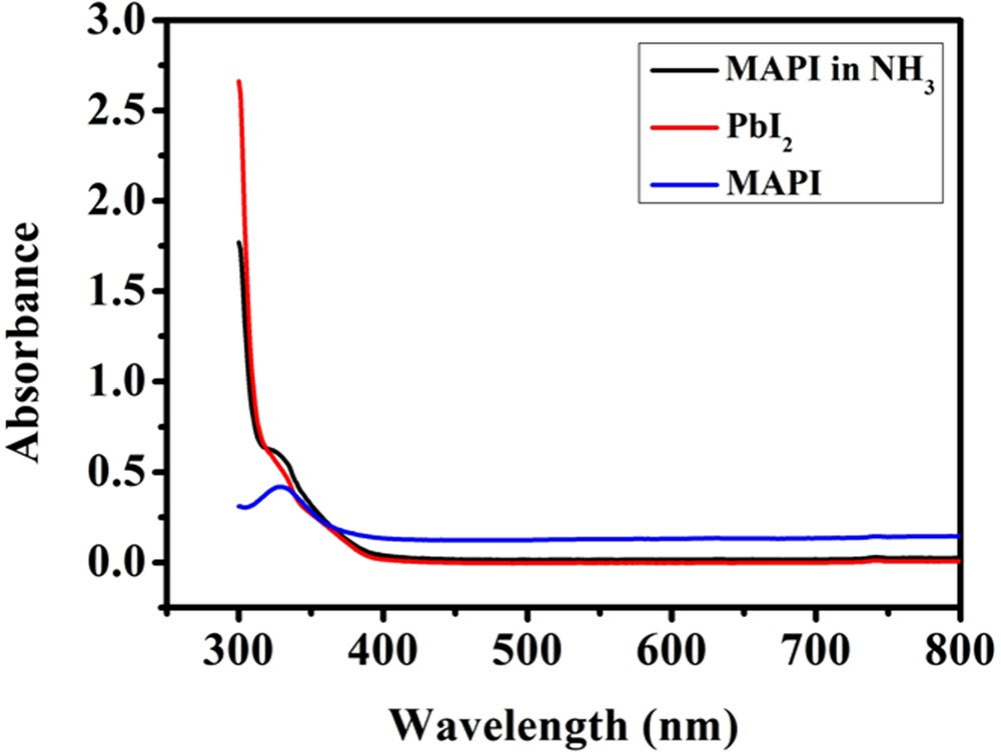
Comparison among UV-VIS absorption of unexposed pristine MAPI on paper, NH_3_ exposed MAPI and PbI_2_ film on paper.

The UV-VIS absorption and the Photo Luminescence (PL) spectra provide further evidence in support of the mechanism proposed for the color change. In Fig. 11 we show the UV-VIS spectra for the pristine MAPI film, the exposed film and the PbI_2_ film. The UV-VIS absorption spectra of the MAPI film in presence of NH_3_ (red line) and that in the pristine film in absence of NH_3_ (black line) are shown.

MAPI film starts to absorb around 750–800 nm and strongly absorbs for wavelength below 500 nm. But in contrast the film exposed to NH_3,_ the absorbance of the film is nearly zero in the visible region and it starts to absorb only in the UV region for wavelength below 400 nm. Comparison with the absorption spectra of the PbI_2_ film shows that the NH_3_ treated MAPI film has similar absorption spectra as that of the PbI_2_ film. This provides a further proof in favor of our hypothesis.

**Figure 12:**
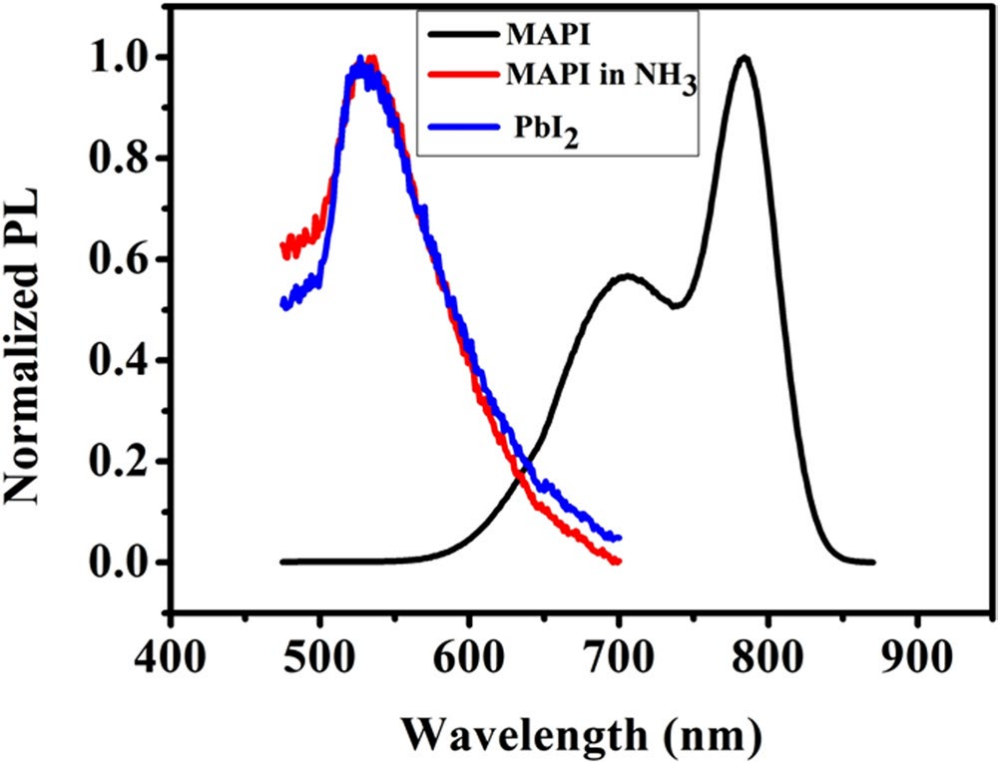
Comparison among PL spectra of of unexposed pristine MAPI on paper, exposed MAPI (to NH_3_) and PbI_2_ film on paper.

We also measured the photoluminescence (PL) spectra of the MAPI film, NH_3_ treated MAPI film and that of a PbI_2_ film as shown in Fig. 12. A comparison of the PL spectra of the NH_3_ treated film with that of the PbI_2_ films shows similarity of the two and again establishing that the dominant phase of the MAPI film on exposure to NH_3_ gas that gives it the distinct color is a PbI_2_ phase.

A series of experiments that establish that the mechanism on which the color change sensor is based on complete degradation of MAPI to PbI_2_, a solid with distinct yellow color, on exposure to NH_3_. The color change to yellow colored PbI_2_ is a structural phase transition that occurs due to interaction of MAPI with ammonia.

From all the experimental observations it has been established that the visual color change based sensor made of MAPI on a cheap paper is disposable type, workable at room temperature and irreversible in nature. The color change corresponds to complete structural change from MAPI to PbI_2_ in an irreversible way.

## Conclusion

In summary, we have demonstrated that a highly selective, disposable and cheap paper sensor based on perovskite halide MAPI can be made to detect presence of the toxic gas NH_3_ by just color change. The black colored MAPI film changes to yellow one in presence of a very low concentration of NH_3_ gas. The sensor can detect presence of NH_3_ gas in open or closed atmosphere down to around 10 ppm. Although some studies discussed about the color change of the MAPI film in presence of NH_3_, but did not mention the concentration of NH_3_ for changing the color^14,15^. The papers mentioned above used the conducting glass (like FTO) as substrate to grow MAPI film. We have used paper (with high porosity) as a substrate. As a result the morphologies of the two films are different and in our case nanorod like structure is responsible for fast response towards NH_3_.The MAPI sensor is easy to fabricate via wet chemistry route and being a visual color change sensor does not need any other extra equipment for its operation. The sensor works at room temperature and shows rapid response that is faster than the response of electrical sensors available for the same gas commercially. We also show the sensor is not sensitive to moisture with RH upto 90% and does not also respond to gases like Methane (CH_4_), Nitrous Oxide (N_2_O), Carbon dioxide (CO_2_) etc in the test chamber each up to a concentration of 500 ppm.

We proposed that conversion of MAPI to PbI_2_ constitutes the mechanism of color change and establish the same using a collection of techniques like XRD, EDX, UV-Visible absorption and Photo Luminescence.

## Experimental Details

### Fabrication of The Gas Sensor with MAPI Paper

The sensor has been fabricated using a simple wet chemistry route. Synthesis of methyl ammonium iodide (CH_3_NH_3_) was done by standard method^16^ by adding Hydro Iodic (HI) acid with ice cooled Methyl Ammonium (CH_3_NH_2_) solution. Details are given in Supplementary notes. The starting step is to make a saturated solution of lead iodide which has been obtained by mixing Lead Iodide in Dimethylformamide (DMF). The solution was spin coated on a commonly used paper at few thousand rpm for about 30 sec. After oven drying the spin coated paper was immersed for 24 hrs in a solution of CH_3_NH_3_I in Iso- Propyl Alcohol (IPA). This leads to formation of MAPI film on the paper. The black colored Methyl Ammonium Lead Iodide (MAPI) coated paper is then dried and it is ready to use.

### Characterizations of The MAPI Film on Paper

**Figure 13:**
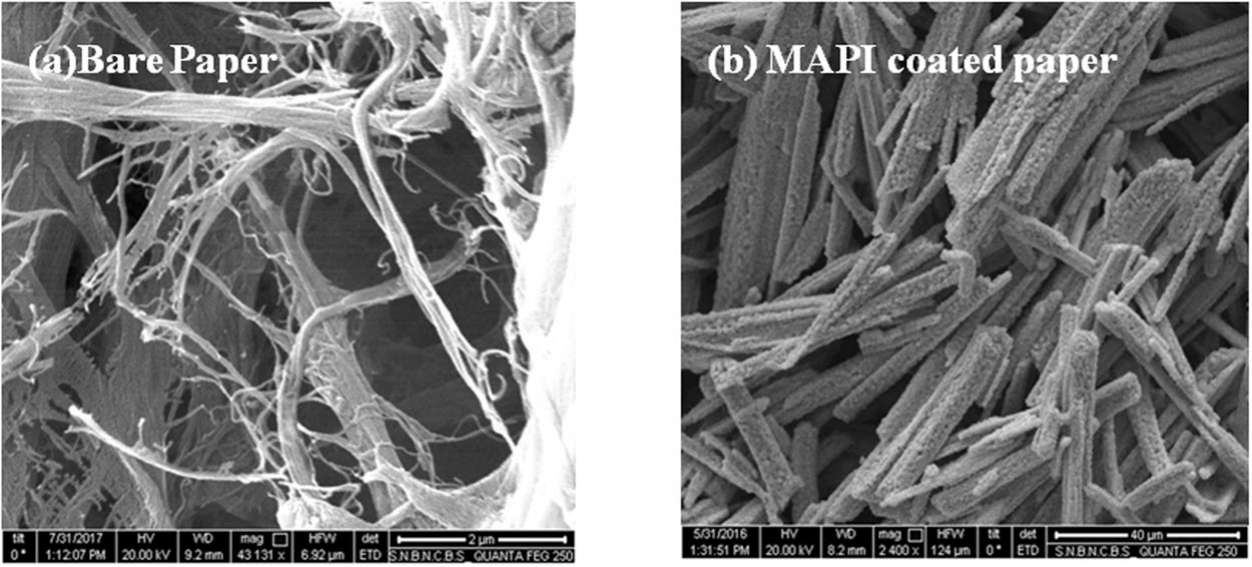
FESEM Images of (**a**) bare paper and (**b**) MAPI thin film grown on paper.

Formation of MAPI film on paper has not been reported before. Below we discuss the characterization of the as grown films by techniques like X-ray diffraction (XRD), Field Emission Scanning Electron Microscope (FESEM) and Energy Dispersive Analysis of X-ray (EDX). FESEM image of the as grown MAPI film on the paper is shown in Fig. 13 along with the image of the bare paper. The bare paper shows fibrous structure arising from cellulose fibers. The MAPI uses these fibers as templates and grows around the cellulose fibers with nanorod like structure. Typical diameter of the MAPI nanorods is in the range of 750 nm and length 40 µm. We find that the formation of these nanorods is important for the performance of the MAPI coated paper as color change sensor.

**Figure 14:**
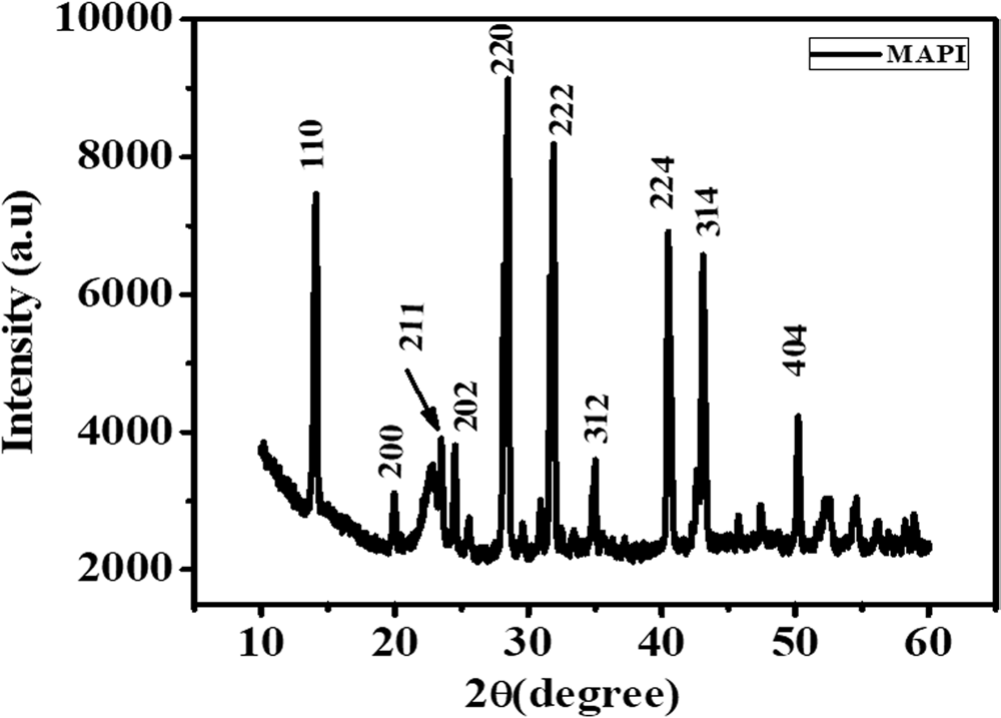
XRD of MAPI film grown on paper.

The phase formation and phase purity of the as grown MAPI nanorods were checked using X-ray diffraction of the MAPI on the paper (which we refer to as MAPI film). The XRD pattern shown in Fig. 14 below can be indexed by tetragonal phase of MAPI and matches well with the ICSD data. (ICSD reference code -250739).

## Electronic supplementary material

Supplementary Information
