# Supplementary material for: Fast response paper based visual color change gas sensor for efficient ammonia detection at room temperature
Source: Sci Rep. 2018 Nov 15;8:16851. doi: 10.1038/s41598-018-33365-3 (PMC6237894; doi:10.1038/s41598-018-33365-3)
Supplement: Supplementary file 1 — Supplementary Information [file 41598_2018_33365_MOESM1_ESM.docx]

Supplementary Section of the manuscript entitled as

**Fast response paper based visual color change gas sensor for efficient ammonia detection at room temperature**

**Avisek Maity^1^, Barnali Ghosh^1,2*^**

*1 Department of Condensed Matter Physics and Materials Sciences, S.N. Bose National Centre for Basic Sciences, JD Block, Sec-III, Salt Lake, Kolkata-700106, India*

*2 Technical Research Centre (TRC), S.N. Bose National Centre for Basic Sciences, JD Block, Sec-III, Salt Lake, Kolkata-700106, India*

*** Corresponding Author

Email: barnali@bose.res.in

**Synthesis of MAI**

There are three major steps involved with the MAI synthesis

1. Synthesis of methyl ammonium iodide provided by the reaction of hydro Iodic (HI) acid with ice cooled methyl ammonium solution.

Initially, HI acid added slowly to ice cooled methyl ammonium solution under stirring for 3-4 hrs until a dark brown solution of methyl ammonium iodide has been formed.

CH_3_NH_2_+HI CH_3_NH_3_I

1. The dark brown colored solution was heated for an hour at 100^o^C and cooled keeping at room temperature. Then cold diethyl ether was added to clean it. A color less crystal forms. The cleaning process using diethyl ether is done repeatedly to achieve the colorless crystal completely from the solution.
2. Recrystallization: The colorless crystals are redissolved in Ethanol and cold ether is added until the whole colorless solution transformed to white precipitation powder. Then the white powder was taken out from filter paper for vacuum annealing (at 60^0^ c for 24 hrs). This was the final product of CH_3_NH_3_I.

Table S1: Different ammonia sensor based on other materials and their response time to detect the ammonia gas concentration

| **NH_3_Sensors** | **Response Time (in sec)** | **NH_3_ gas Concentration (in ppm)** |
| --- | --- | --- |
| rGo | 108 | 10 [1] |
| PANI | 800 | 50 [2] |
| SNO_2_ | 3000 | 10 [3] |
| CNT –TiO_2_ | 600 | Not mentioned [4] |

***References:***

[1] Tai, H., Yuan, Z., Zheng, W. , Ye, Z., Liu , C. , Du ,X. ZnO Nanoparticles/Reduced Graphene Oxide Bilayer Thin Films for Improved NH_3_-Sensing Performances at Room Temperature; Nano Express, (2016) DOI 10.1186/s11671-016-1343-7.

[2] Kumar , L. , Rawal , I. , Kaur , A. , Annapoorni, S. Flexible room temperature ammonia sensor based on polyaniline; Sensors and Actuators B **240**, 408–416(2017).

[3] Samà , J. et al. Site-selectively grown SnO_2_ NWs networks on micromembranes for efficient ammonia sensing in humid conditions; Sensors and Actuators B **232**, 402–409 (2016) .

[4] Sánchez , M. , Rincón, M. Ammonia Sensors Based on Composites of Carbon Nanotubes and Titanium Dioxide; Carbon Nanotubes – Growth and Applications; **18**,458-470.
